# *Matrix metalloproteinase-9* Gene-1562C>T Gene Polymorphism and Coronary Artery Disease in the Chinese Han Population: A Meta-Analysis of 5468 Subjects

**DOI:** 10.3389/fphys.2016.00212

**Published:** 2016-06-09

**Authors:** Yan-Yan Li, Xin-Xing Yang, Yan-Hong Zhou, Ge Gong, Hong-Yu Geng, Hyun J. Kim, Chuan-Wei Zhou, Yun Qian, Xiang-Ming Wang, Jun Wu

**Affiliations:** ^1^Department of Geriatrics, First Affiliated Hospital of Nanjing Medical UniversityNanjing, China; ^2^Department of Radiation Oncology, University of PennsylvaniaPhiladelphia, PA, USA

**Keywords:** *matrix metalloproteinase-9*, -1562C>T, polymorphism, coronary artery disease, Chinese

## Abstract

**Background:** Multiple studies indicate that the *matrix metalloproteinase-9 (MMP-9)*-1562C>T gene polymorphism may be associated with an increased risk of coronary artery disease (CAD) in the Chinese Han population. However, a clear consensus has yet to be established.

**Objective and methods:** A meta-analysis of 5468 subjects from 10 separate studies was performed to explore the possible relationship between the *MMP-9*-1562C>T gene polymorphism and CAD within the Chinese Han population. Pooled odds ratio (ORs) for the association and the corresponding 95% confidence intervals (CIs) were evaluated by a random or fixed-effect model.

**Results:** Our analysis confirms the association between the *MMP-9*-1562C>T gene polymorphism and an increased risk of CAD within the Chinese Han population under allelic (OR: 1.60, 95% CI: 1.25–2.04, *P* = 0.0002), recessive (OR: 3.05, 95% CI: 1.67–5.56, P = 0.0003), dominant (OR: 2.23, 95% CI: 1.49–3.35, *P* = 0.0001), homozygous (OR: 3.41, 95% CI: 1.87–6.23, *P* < 0.0001), heterozygous (OR: 2.03, 95% CI: 1.40–2.93, *P* = 0.0002), and additive genetic models (OR: 1.78, 95% CI: 1.33–2.39, *P* < 0.0001).

**Conclusions:** In the Chinese Han population, the *MMP-9*-1562C>T gene polymorphism is correlated with an increased risk of CAD. Therefore, Han Chinese carriers of the -1562T allele may be at an increased risk of CAD.

## Introduction

Coronary artery disease (CAD) is a chronic condition with both hereditary and environmental factors. The increasing prevalence of unhealthy lifestyles in China (i.e., extended periods of sitting, a more sedentary lifestyle, a high-fat diet), the general aging of the Chinese population, and the increasing rates of hypertension and diabetes have damaged the quality of life of many Chinese people and sharply increased CAD-associated morbidity and mortality (Li, 2007). However, recent advancements in molecular biology have allowed researchers to better elucidate the pathogenic mechanism of CAD.

Matrix metalloproteinases (MMPs), a large family of zinc-dependent proteolytic enzymes that have special functions in extracellular matrix (ECM) degradation have been a particular rich area of research. MMPs play a role in multiple pathophysiological processes such as ECM degradation, inflammation response, tumor metastasis, and atherosclerosis (Castro and Tanus-Santos, 2013). Enhanced MMP expression and activity also play a pivotal role in early arteriosclerosis, plaque rupture, myocardial infarction, and heart failure (Tanner et al., 2011). *MMP-9* (also known as gelatinase B) is one the most extensively researched MMPs and exhibits enhanced expression in atherosclerosis injury sites. It has been also found that there were many gene sequence variations in *MMP-9* gene, of which the locus rs3918242 (-1562C>T) in the promoter region was the most reported gene locus.

*MMP-9* gene, located in 20q11.2–13.1, spans 7.7 kb and contains 13 exons. *MMP-9*-1562C>T mutation occurs in a substitution of cytosine (C) for thymine (T). This mutation may increase *MMP-9* gene expression and risk of CAD development by reducing the binding affinity with transcription inhibition proteins.

Although many studies on the association between *MMP-9*-1562C>T gene polymorphism and CAD have been performed in China, the composite of these studies fail to provide a consensus. In 2005, Tang et al found that *MMP-9*-1562C>T gene polymorphism was significantly associated with CAD in a Zhejiang population and T allele increased the CAD risk (Tang et al., 2005). In 2007, Chen et al also reported a similar result in another Hunan population (Chen et al., 2007). In contrast, Wu et al failed to find an association between *MMP-9*-1562C>T gene polymorphism and CAD in a Beijing population in 2009 (Wu et al., 2009) and Zhi et al observed no significant effects for *MMP-9*-1562C>T gene polymorphism on CAD risk in a Jiangsu population (Zhi et al., 2010).

We performed the current meta-analysis with the hopes to provide a valuable conclusion on the association between *MMP-9*-1562C>T gene polymorphism and CAD in the Chinese Han population.

## Materials and methods

### Publication search and inclusion criteria

The Web of Science, PubMed, Embase, the China Biological Medicine Database, and the China National Knowledge Infrastructure electronic databases were searched using the terms “*matrix metalloproteinase-9*,” “coronary artery disease,” or “coronary heart disease,” and “polymorphism” in our initial search. Retrieved studies were published between 2005 and 2010 with the last study updated on March 26, 2016.

To meet our inclusion criteria, studies had to (a) evaluate the association between *MMP-9*-1562C>T gene polymorphism and CAD in the Chinese Han population. (b) diagnose CAD according to the clinical symptoms combined with examination results (i.e., coronary arteriography, electrocardiogram, treadmill exercise test, echocardiography, myocardial perfusion imaging by Emission Computed Tomography, etc.) with a minimal stenosis rate of the major coronary artery diameter of more than 50% (c) be a case-control or cohort study published in an official journals or as a postgraduate dissertation.

### Data extraction

Data was extracted according to a standardized protocol. Two investigators searched for duplicates while a third served as an arbiter to resolve possible disagreements. Duplicate papers, those that violated the inclusion criteria, or those that provided deficient data were removed. Identical data sets used in different studies by the same authors were used once. Abstracted data consisted of the following items: the first author's name, publication year, region, number of genotypes, genotyping method, study design, age, gender, and total number of cases and controls.

### Statistical analysis

The odds ratio (OR) and its corresponding to 95% confidence interval (CI) were used to measure the association between *MMP-9*-1562C>T gene polymorphism and CAD. The Chi-square-based Q-test was adopted to measure the effects between-studies heterogeneity (*P* < 0.05 level) (Cochran, 1968). Variation due to heterogeneity was assessed by the inconsistency index *I*^2^. If heterogeneity were present in the study, the random-effects model would be used to assess the combined OR (the DerSimonian and Laird method) (DerSimonian and Laird, 1986). Otherwise, the fixed-effects model would be used (the Mantel-Haenszel method) (Mantel and Haenszel, 1959). The pooled OR was determined by *Z*-test and significance was set at *P* < 0.05.

The Fisher's exact test was used to evaluate the Hardy-Weinberg equilibrium (HWE) (*P* < 0.05). Potential publication bias was assessed by Egger′s linear regression test on the natural log scale of the OR to detect funnel plot asymmetry (*P* < 0.05 level) (Egger et al., 1997). The statistical analysis was performed by STATA 11.0 software (StataCorp, College Station, TX).

## Results

### Studies and populations

Ten of the nineteen retrieved papers fit the inclusion criteria. Of the nine excluded studies, one was a duplicate, four were reviews, and another four were irrelevant to our interests. No study was excluded for deviation from HWE. The ten studies compiled the data from 3168CAD patients and 2300 controls (Table 1, Presentation 1 in Supplementary Material; Tang et al., 2005; Meng et al., 2006; Chen et al., 2007; Wang et al., 2007; Wu et al., 2009; Gao and Wang, 2010; Ma et al., 2010; Yong and Shi, 2010; Zhang et al., 2010; Zhi et al., 2010) and represented seven provinces (Shanxi, Xinjiang, Jiangsu, Zhejiang, Hunan, Tianjin, and Beijing). All subjects were of Han ethnicity. However, there were substantial differences in the size of the total patient population between these studies. The Wu N study alone contributed 1356 CAD patients and 689 controls for this meta-analysis. Differences in patient number may be factor in the lack of consensus on this topic.

**Table 1:** **Characteristics of the investigated studies of the association between the ***matrix metalloproteinase-9*** gene -1562C>T polymorphism and coronary artery disease in the Chinese population**.

**Author**	**Year**	**Region**	**CAD**			**Control**			**Age (years old)**		**Gender (female/male)**		**Sample size (CAD/control)**
			**CC**	**CT**	**TT**	**CC**	**CT**	**TT**	**CAD**	**Control**	**CAD**	**Control**	
Tang et al.	2005	Zhejiang	73	27	1	91	13	1	64.1±10.7	62.3±11.2	73/28	86/19	101/105
Meng et al.	2006	Tianjin	91	26	0	80	18	1	56.4±7.7	54.3±8.1	40/77	32/67	117/99
Chen et al.	2007	Hunan	63	25	2	58	11	1	59.2±10.4	57.7±10.2	57/33	44/26	90/70
Wang et al.	2007	Shanxi	46	17	1	66	18	0	63.08±13.3	62.55±9.2	43/21	52/32	64/84
Wu et al.	2009	Beijing	1078	263	15	545	143	1	58.39±10.84^*^	60.42±9.07	1162/194	589/100	1356/689
Gao and Wang	2010	Jiangsu	49	38	9	59	18	1	64.45±9.88	62.74±9.17	59/37	45/33	96/78
Ma et al.	2010	Xinjiang	266	84	12	348	67	4	56.7±9.4	55.2±10.1	249/113	292/127	362/419
Yong and Shi	2010	Zhejiang	97	30	1	92	14	0	64.0±10.47	61.0±11.22	74/54	65/41	128/106
Zhang et al.	2010	Shanxi	67	22	3	83	12	0	54.50±6.4	52.30±6.9	62/30	59/36	92/95
Zhi et al.	2010	Jiangsu	585	174	3	442	110	3	67.46±9.61	69.90±11.48	543/219	372/183	762/555

*CAD, coronary artery disease*.

*Polymerase chain reaction-restriction fragment length polymorphism genotyping method and Case-control study design were adopted in the above studies*.

*Compared with control group*,

* *P < 0.05*.

### Combined analyses

**Figure 1:**
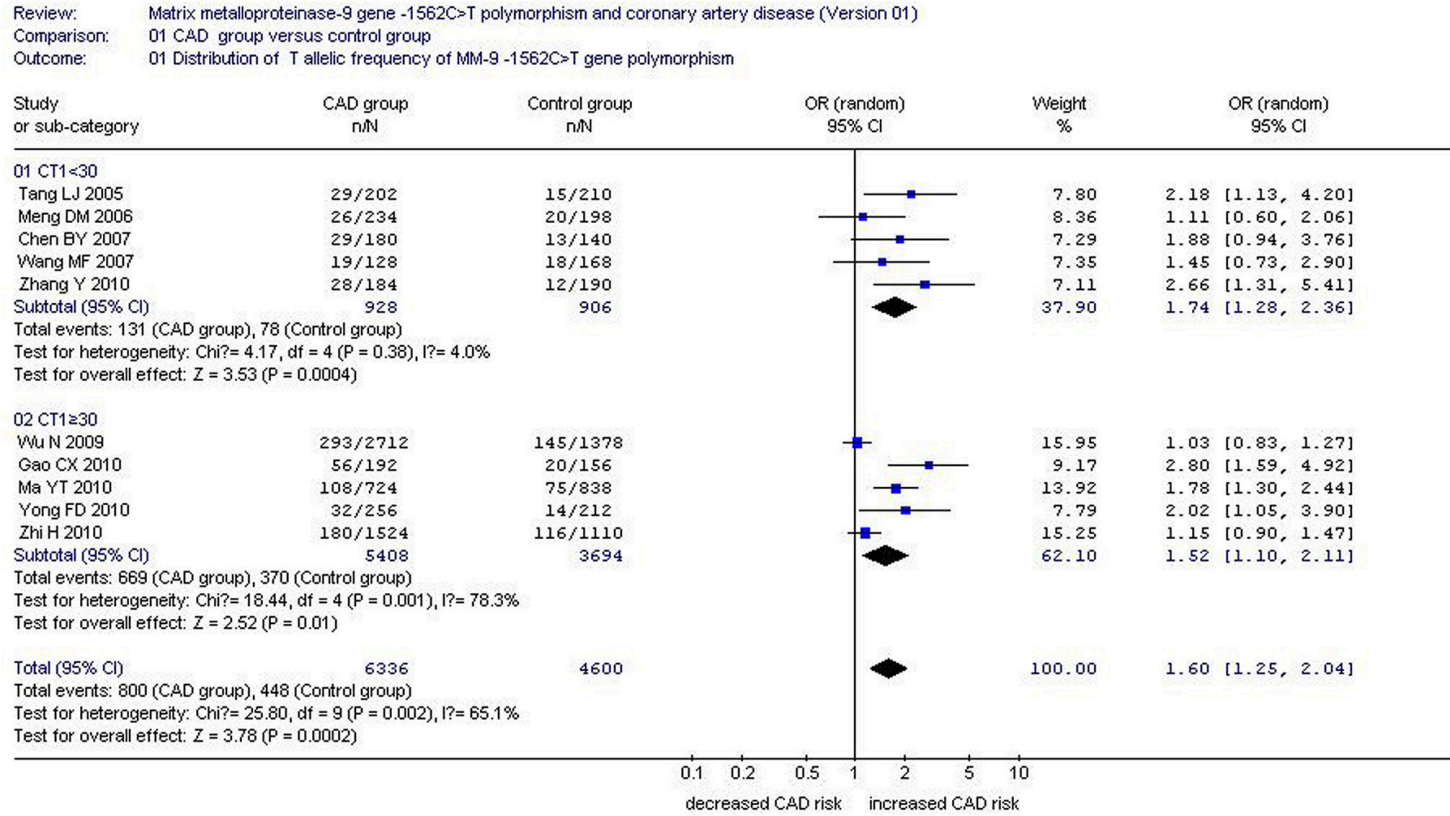
**Forest plot of coronary artery disease associated with ***MMP-9***-1562C>T gene polymorphism under an allelic genetic model stratified by CT1 (distribution of T allelic frequency of ***MMP-9*** gene)**.

**Figure 2:**
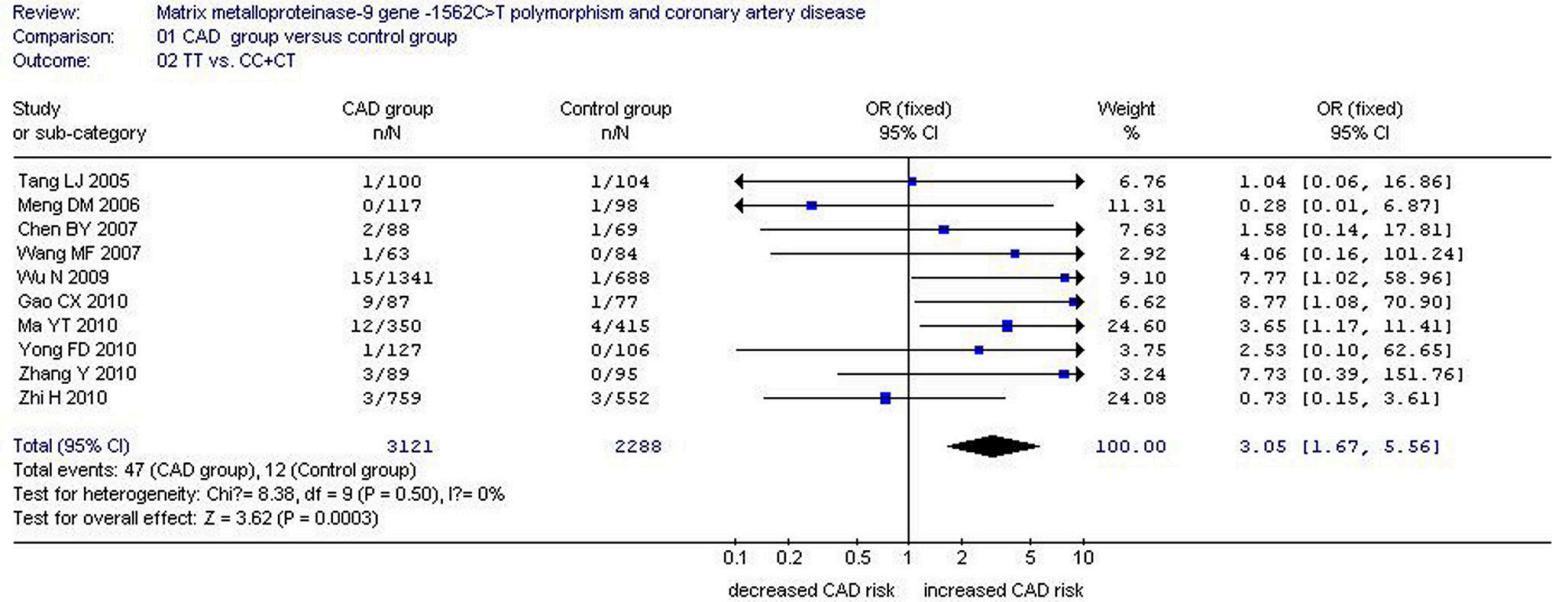
**Forest plot of coronary artery disease associated with ***MMP-9***-1562C>T gene polymorphism under a recessive genetic model (TT vs. CC+CT)**.

**Figure 3:**
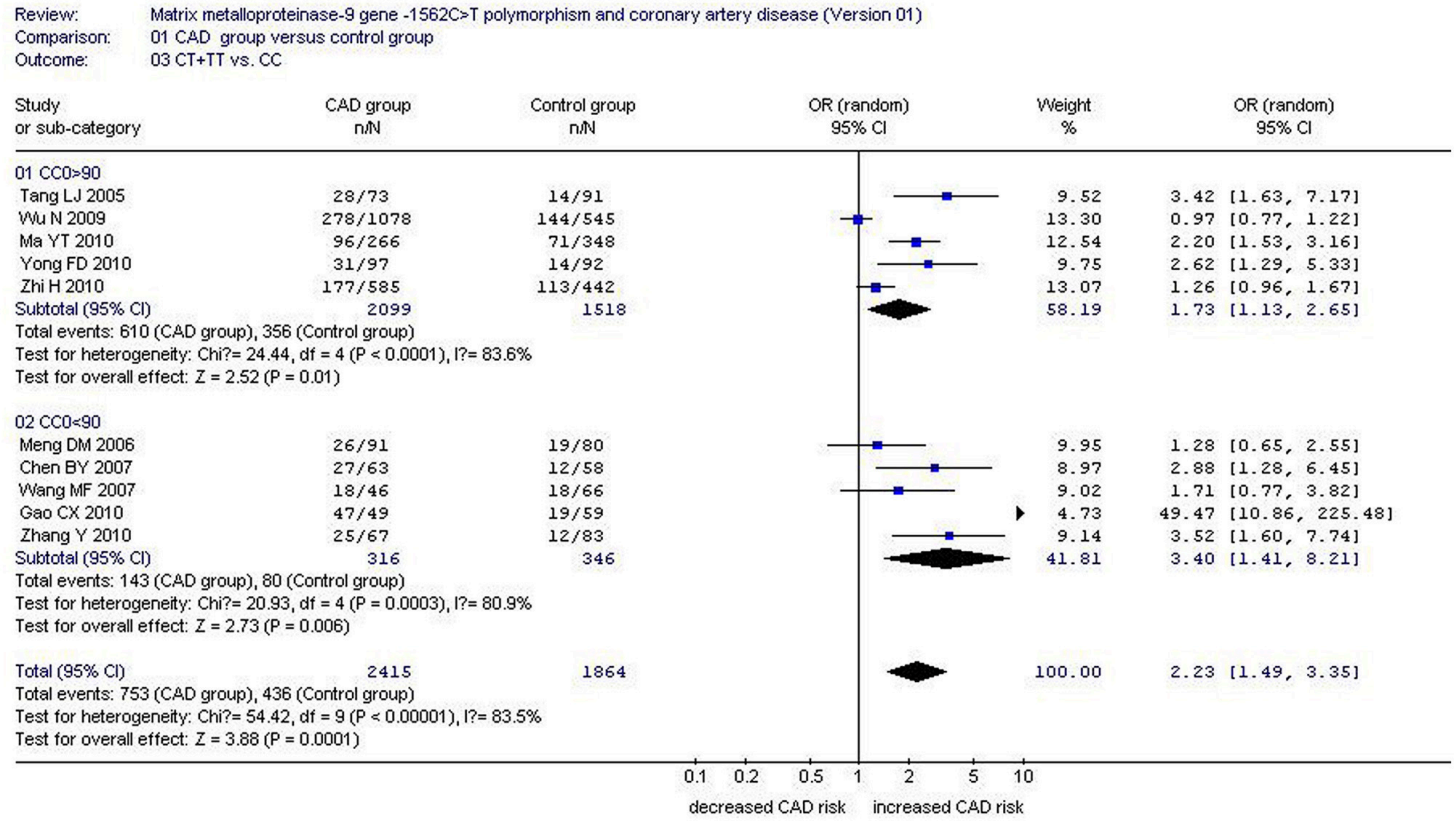
**Forest plot of coronary artery disease associated with ***MMP-9***-1562C>T gene polymorphism under a dominant genetic model stratified by CC0 (CT+TT vs. CC)**.

**Figure 4:**
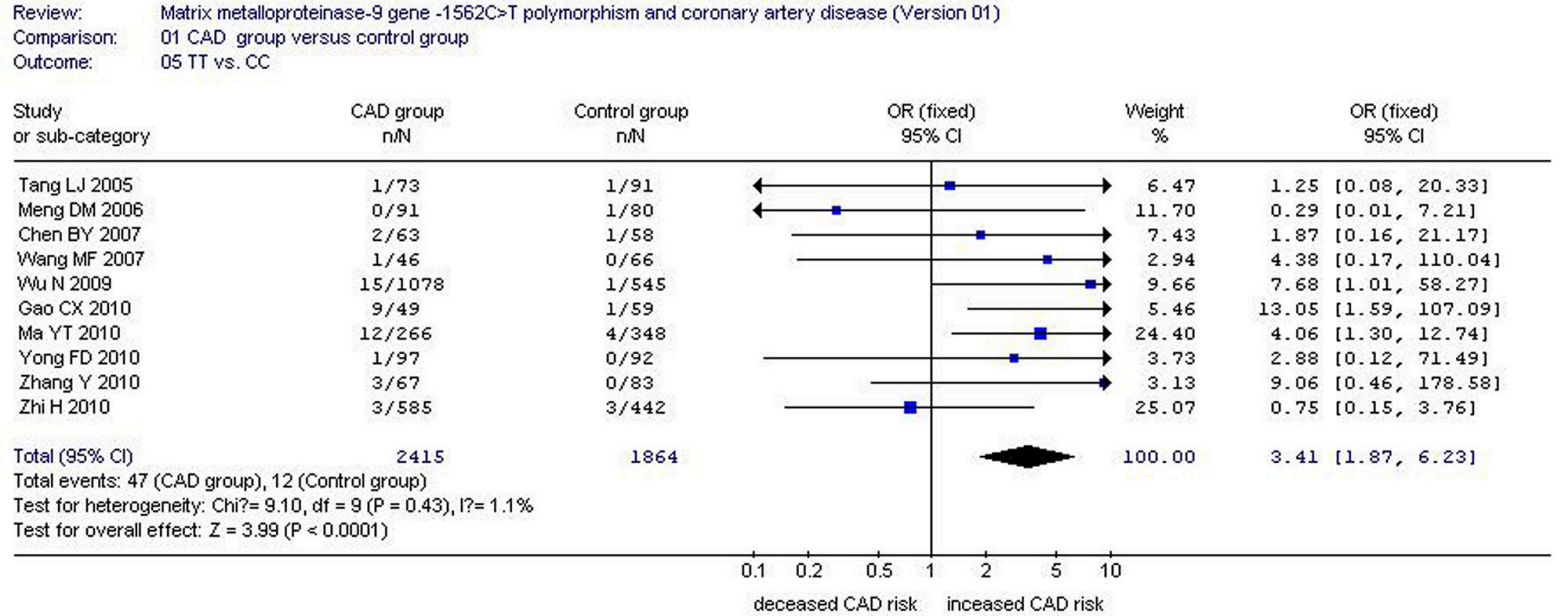
**Forest plot of coronary artery disease associated with ***MMP-9***-1562C>T gene polymorphism under a homozygous genetic model (TT vs. CC)**.

**Figure 5:**
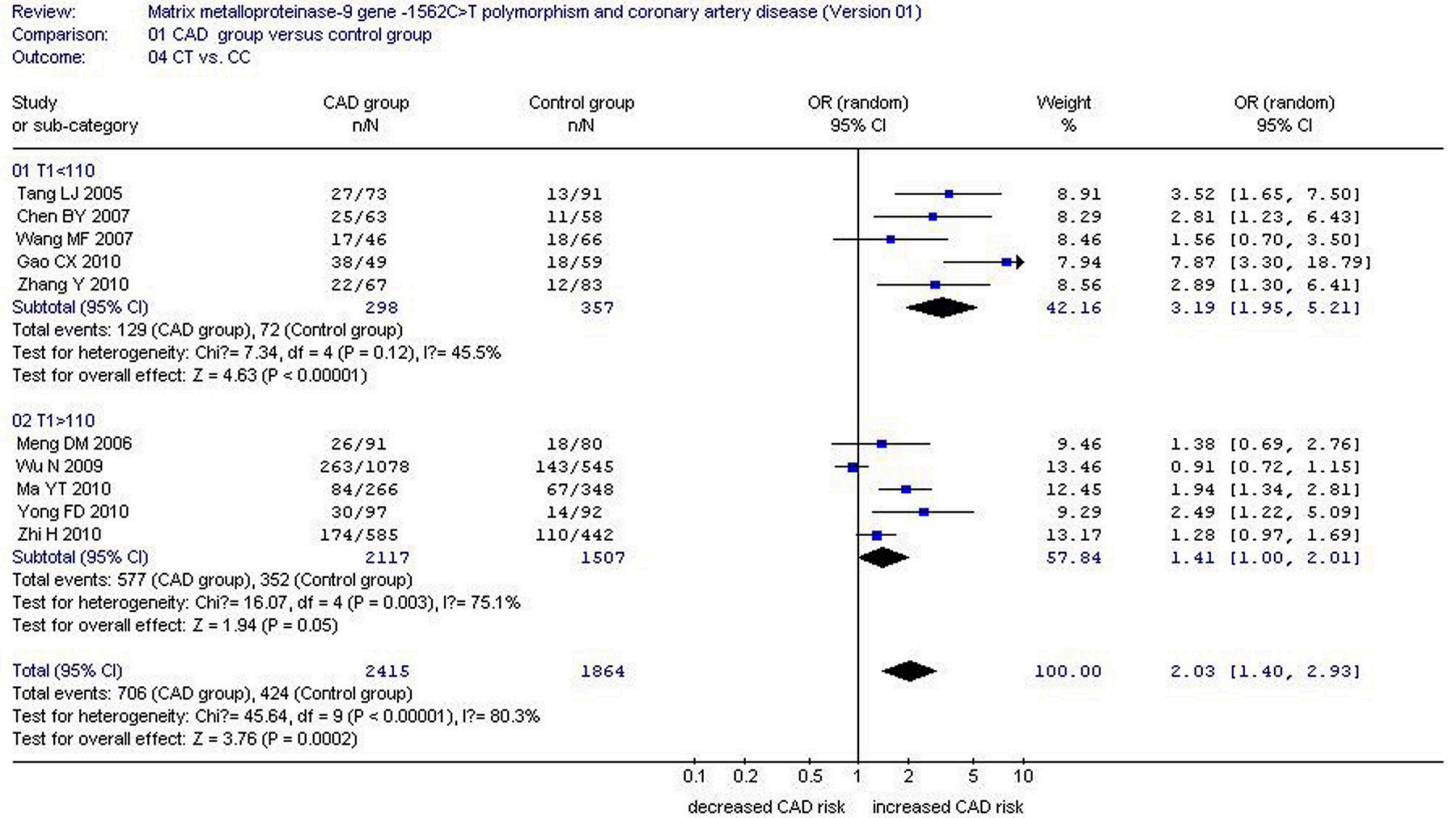
**Forest plot of coronary artery disease associated with ***MMP-9***-1562C>T gene polymorphism under a heterozygous genetic model stratified by T1 (CT vs. CC)**.

**Figure 6:**
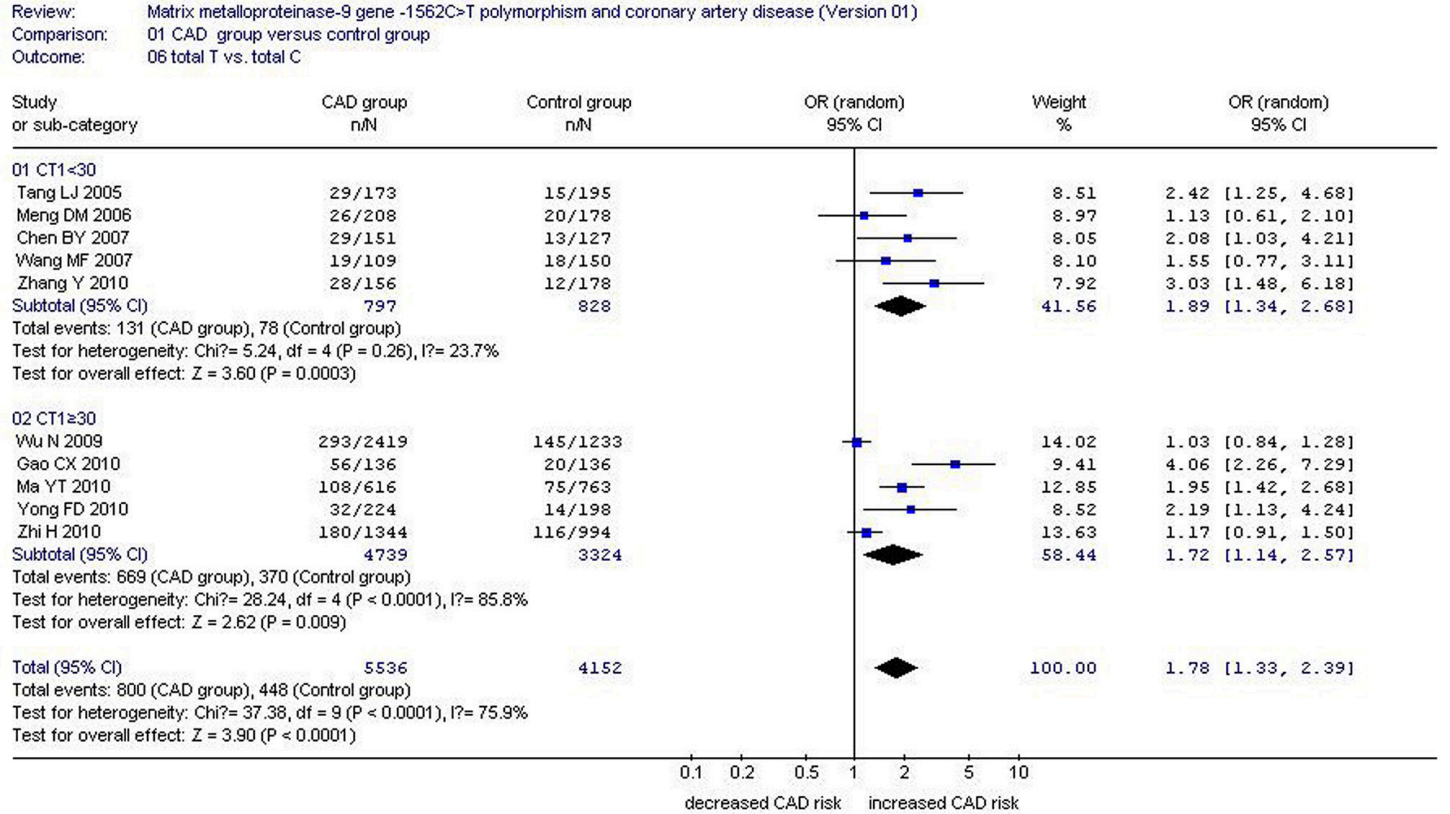
**Forest plot of coronary artery disease associated with ***MMP-9***-1562C>T gene polymorphism under an additive genetic model stratified by CT1 (Total T vs. Total C)**.

There was a significant association between *MMP-9*-1562C>T gene polymorphism and CAD in the Chinese Han population under allelic (OR: 1.60, 95% CI: 1.25–2.04, *P* = 0.0002), recessive (OR: 3.05, 95% CI: 1.67–5.56, *P* = 0.0003), dominant (OR: 2.23, 95% CI: 1.49–3.35, *P* = 0.0001), homozygous (OR: 3.41, 95% CI: 1.87–6.23, *P* < 0.0001), heterozygous (OR: 2.03, 95% CI: 1.40–2.93, *P* = 0.0002), and additive genetic models (OR: 1.78, 95% CI: 1.33–2.39, *P* < 0.0001). (Table 2, Figures 1–6).

**Table 2:** **Summary of meta-analysis of association of ***matrix metalloproteinase-9*** gene -1562C>T polymorphism and coronary artery disease in the Chinese population**.

**Genetic model**	**Pooled OR (95% CI)**	***P*-value**	**Literature number**	**CAD size**	**Control size**	***P*_heterogeneity_ (*I*^2^%)**
Allelic genetic model	1.60 (1.25–2.04)	0.0002^*^	10	3168	2300	0.002^*^(65.1%)
Subgroup 1: CT1 < 30	1.74 (1.28–2.36)	0.0004^*^	5	464	453	0.38(4.0%)
Subgroup 2: CT1≥30	1.52 (1.10–2.11)	0.01^*^	5	2704	1847	0.001^*^(78.3%)
Recessive genetic model	3.05 (1.67–5.56)	0.0003^*^	10	3168	2300	0.50(0%)
Dominant genetic model	2.23 (1.49–3.35)	0.0001^*^	10	3168	2300	< 0.00001^*^(83.5%)
Subgroup 1: CC0>90	1.73 (1.13–2.65)	0.01^*^	5	2709	1874	< 0.0001^*^(83.6%)
Subgroup 2: CC0 < 90	3.40 (1.41–8.21)	0.006^*^	5	459	426	0.0003^*^(80.9%)
Homo genetic model	3.41 (1.87–6.23)	< 0.0001^*^	10	3168	2300	0.43(1.1%)
Hetero genetic model	2.03 (1.40–2.93)	0.0002^*^	10	3168	2300	< 0.00001^*^(80.3%)
Subgroup 1: T1 < 110	3.19 (1.95–5.21)	< 0.00001^*^	5	443	432	0.12(45.5%)
Subgroup 2: T1>110	1.41 (1.00–2.01)	0.05^*^	5	2725	1868	0.003^*^(75.1%)
Additive genetic model	1.78 (1.33–2.39)	< 0.0001^*^	10	3168	2300	< 0.0001^*^(75.9%)
Subgroup 1: CT1 < 30	1.89 (1.34–2.68)	0.0003^*^	5	464	453	0.26(23.7%)
Subgroup 2: CT1≥30	1.72 (1.14–2.57)	0.009^*^	5	2704	1847	< 0.0001^*^(85.8%)

* *P < 0.05*.

*CAD, coronary artery disease; CI, confidence interval; OR, odds ratio; CAD size, the total number of CAD cases; control size, the total number of control group; homo genetic model, homozygous genetic model; hetero genetic model, heterozygous genetic model; CT1, CT sample size of CAD group; CC0, CC sample size of control group; T1, Total sample size of CAD group*.

There was also significant heterogeneity under the allelic (*P* = 0.002, *I*^2^ = 65.1%), dominant (*P* < 0.00001, *I*^2^ = 83.5%), heterozygous (*P* < 0.00001, *I*^2^ = 80.3%) and additive genetic models (*P* < 0.0001, *I*^2^ = 75.9%). Subsequent meta-regressions explored the source of this heterogeneity under their respective genetic models.

Under the allelic genetic model, CC sample size of CAD group (CC1, *P* = 0.006), CT sample size of CAD group (CT1, *P* = 0.005), TT sample size of CAD group (TT1, *P* = 0.010), CC sample size of control group (CC0, *P* = 0.023), total sample size of control group (T0, *P* = 0.034), and weight (%) (*P* = 0.030)were possible confounding factors that could partially explain heterogeneity between studies.

Under the additive genetic model, CC1 (*P* = 0.013), CT1 (*P* = 0.012), and TT1 (*P* = 0.016) were the confounding factors. In the allelic and additive genetic models, CT1 plays a central role in explaining the source of heterogeneity. According to CT1, the whole population was separated into two subgroups. The studies with CT1 < 30 were grouped into subgroup 1 and the residual studies with CT1 ≥ 60 belonged to subgroup 2.

Stratified by CT1, a subgroup analysis under allelic and additive genetic models found significant association between *MMP-9*-1562C>T gene polymorphism and CAD in both subgroups (allelic genetic model: subgroup 1: OR: 1.74, 95% CI: 1.28–2.36, *P* = 0.0004; subgroup 2: OR: 1.52, 95% CI: 1.10–2.11, *P* = 0.01) (additive genetic model: subgroup 1: OR: 1.89, 95% CI: 1.34–2.68, *P* = 0.0003; subgroup 2: OR: 1.72, 95% CI: 1.14–2.57, *P* = 0.009). No significant heterogeneity was fond in subgroup 1 (allelic genetic model: P_heterogeneity_ = 0.38, *I*^2^ = 4.0%; additive genetic model: P_heterogeneity_ = 0.26, *I*^2^ = 23.7%), but significant heterogeneity was detected in subgroup 2 (allelic genetic model: P_heterogeneity_ = 0.001, *I*^2^ = 78.3%; additive genetic model: P_heterogeneity_ < 0.0001, *I*^2^ = 85.8%). This identifies CT1 as the primary confounding factor under the allelic and additive genetic models (Tables 2−4; Figures 1, 6).

**Table 3:** **The meta-regression results among 10 studies in the Chinese population under an allelic genetic model for ***matrix metalloproteinase-9*** gene -1562C>T gene polymorphism**.

	**Coefficient**	**Standard Error**	***T*-value**	***P*-value**	**95% confidence interval**
CC sample size of CAD group	−0.0081427	0.0011763	−6.92	0.006^*^	−0.0118862~−0.0043991
CT sample size of CAD group	0.0405205	0.0054126	7.49	0.005^*^	0.0232951~0.0577458
TT sample size of CAD group	0.0561608	0.009485	5.92	0.010^*^	0.0259754~0.0863462
CC sample size of control group	0.0423375	0.0098008	4.32	0.023^*^	0.0111471~0.0735279
Total sample size of control group	−0.0337639	0.0090819	−3.72	0.034^*^	−0.0626665~−0.0048613
Weight(%)	−0.2944788	0.0755127	−3.90	0.030^*^	−0.534794~−0.0541637
Cons	2.19458	0.4613965	4.76	0.018^*^	0.7262105~3.662949

* *P < 0.05*.

*Coefficient: regression coefficient. The regression coefficients are the estimated increase in the lnOR per unit increase in the covariates. Cons, constant item*.

**Table 4:** **The meta-regression results among 10 studies in the Chinese population under an additive genetic model for ***matrix metalloproteinase-9*** gene -1562C>T gene polymorphism**.

	**Coefficient**	**Standard Error**	***T*-value**	***P*-value**	**95% confidence interval**
CC sample size of CAD group	−0.0091723	0.0017378	−5.28	0.013^*^	−0.0147029~−0.0036417
CT sample size of CAD group	0.0470359	0.0085234	5.52	0.012^*^	0.0199107~0.0741611
TT sample size of CAD group	0.0699275	0.0141987	4.92	0.016^*^	0.0247408~0.1151142
CC sample size of control group	0.007531	0.0022575	3.34	0.045^*^	0.0003466~0.0147153
CT sample size of control group	−0.0376383	0.0137834	−2.73	0.072	−0.0815034~0.0062267
Weight	−0.4013957	0.139735	−2.87	0.064	−0.8460947~0.0433033
Cons	3.40343	1.011299	3.37	0.044^*^	0.1850261~6.621835

* *P < 0.05*.

*Coefficient: regression coefficient. The regression coefficients are the estimated increase in the lnOR per unit increase in the covariates. Cons, constant item*.

Under the dominant genetic model, CC1 (*P* = 0.015), CT1 (*P* = 0.009), TT1 (*P* = 0.009), CC sample size of control group (CC0, *P* = 0.001), CT sample size of control group (CT0, *P* = 0.001), TT sample size of control group (TT0, *P* = 0.012), and weight (*P* = 0.001) could partly explain the heterogeneity source. In a subgroup analysis stratified by CC0, significant increased CAD risk was observed in both subgroups (subgroup 1: OR: 1.73, 95% CI: 1.13–2.65, *P* = 0.01; subgroup 2: OR: 3.40, 95% CI: 1.41–8.21, *P* = 0.006). Although heterogeneity was still detected in both subgroups, it was significantly reduced in subgroup 2 (P_heterogeneity_ = 0.0003, *I*^2^ = 80.9%), suggesting that CC0 was the main confounding factor (Tables 2, 5; Figure 3).

**Table 5:** **The meta-regression results among 10 studies in the Chinese population under a dominant genetic model for ***matrix metalloproteinase-9*** gene -1562C>T gene polymorphism**.

	**Coefficient**	**Standard Error**	***T*-value**	***P*-value**	**95% confidence interval**
Study Region	−0.0108937	0.0011565	−9.42	0.067	−0.0255888~0.0038014
CC sample size of CAD group	−0.0018324	0.0000437	−41.90	0.015^*^	−0.002388~−0.0012768
CT sample size of CAD group	0.016394	0.0002299	71.31	0.009^*^	0.0134728~0.0193151
TT sample size of CAD group	−0.0231394	0.0003334	−69.40	0.009^*^	−0.0273758~−0.018903
CC sample size of control group	0.0214946	0.0000441	487.42	0.001^*^	0.0209343~0.022055
CT sample size of control group	−0.0753318	0.0001097	−686.43	0.001^*^	−0.0767263~−0.0739374
TT sample size of control group	0.0395787	0.0007278	54.38	0.012^*^	0.0303313~0.0488261
Weight	−0.7719044	0.0014531	−531.20	0.001^*^	−0.7903681~−0.7534408
Cons	7.286745	0.0136083	535.46	0.001^*^	7.113836~7.459655

* *P < 0.05*.

*Coefficient: regression coefficient. The regression coefficients are the estimated increase in the lnOR per unit increase in the covariates. Cons, constant item*.

Under the heterozygous genetic model, CC1 (*P* = 0.006), total sample size of CAD group (T1, *P* = 0.011), and CT0 (*P* = 0.004) could partly explain the heterogeneity. In a subgroup analysis stratified by T1, significant increase in CAD risk was detected in both subgroups (subgroup 1: OR: 3.19, 95% CI: 1.95–5.21, *P* < 0.00001; subgroup 2: OR: 1.41, 95% CI: 1.00–2.01, *P* = 0.05). No significant heterogeneity existed in subgroup 1 any longer (P_heterogeneity_ = 0.12, *I*^2^ = 45.5%), but significant heterogeneity was still observed in subgroup 2 (P_heterogeneity_ < 0.0001, *I*^2^ = 85.8%; Tables 2, 6; Figure 5).

**Table 6:** **The meta-regression results among 10 studies in the Chinese population under a heterozygous genetic model for ***matrix metalloproteinase-9*** gene -1562C>T gene polymorphism**.

	**Coefficient**	**Standard Error**	***T*-value**	***P*-value**	**95% confidence interval**
CC sample size of CAD group	−0.0710505	0.013625	−5.21	0.006^*^	−0.1088794~−0.0332215
Total sample size of CAD group	0.0614264	0.013634	4.51	0.011^*^	0.0235723~0.0992805
CT sample size of control group	−0.0498063	0.0082049	−6.07	0.004^*^	−0.0725867~−0.0270259
Cons	0.4150886	0.3419184	1.21	0.292	−0.534229~1.364406

* *P < 0.05*.

*Coefficient: regression coefficient. The regression coefficients are the estimated increase in the lnOR per unit increase in the covariates. Cons, constant item*.

### Sensitivity analysis

In the current meta-analysis, the sensitivity analysis was performed. After the Wu et al. was removed from the current meta-analysis, the association between *MMP-9*-1562C>T gene polymorphism and CAD was further strengthened under the allelic genetic model (OR: 1.72, 95% CI: 1.35–2.19, *P* = 1.3 × 10^−5^, P_heterogeneity_ = 0.04, *I*^2^ = 50.0%; Wu et al., 2009). Removal of other studies respectively from the current studies did not change the results from the original analysis. Hence, the Wu et al study should be the high sensitivity study in the current meta-analysis.

### Bias diagnostics

**Figure 7:**
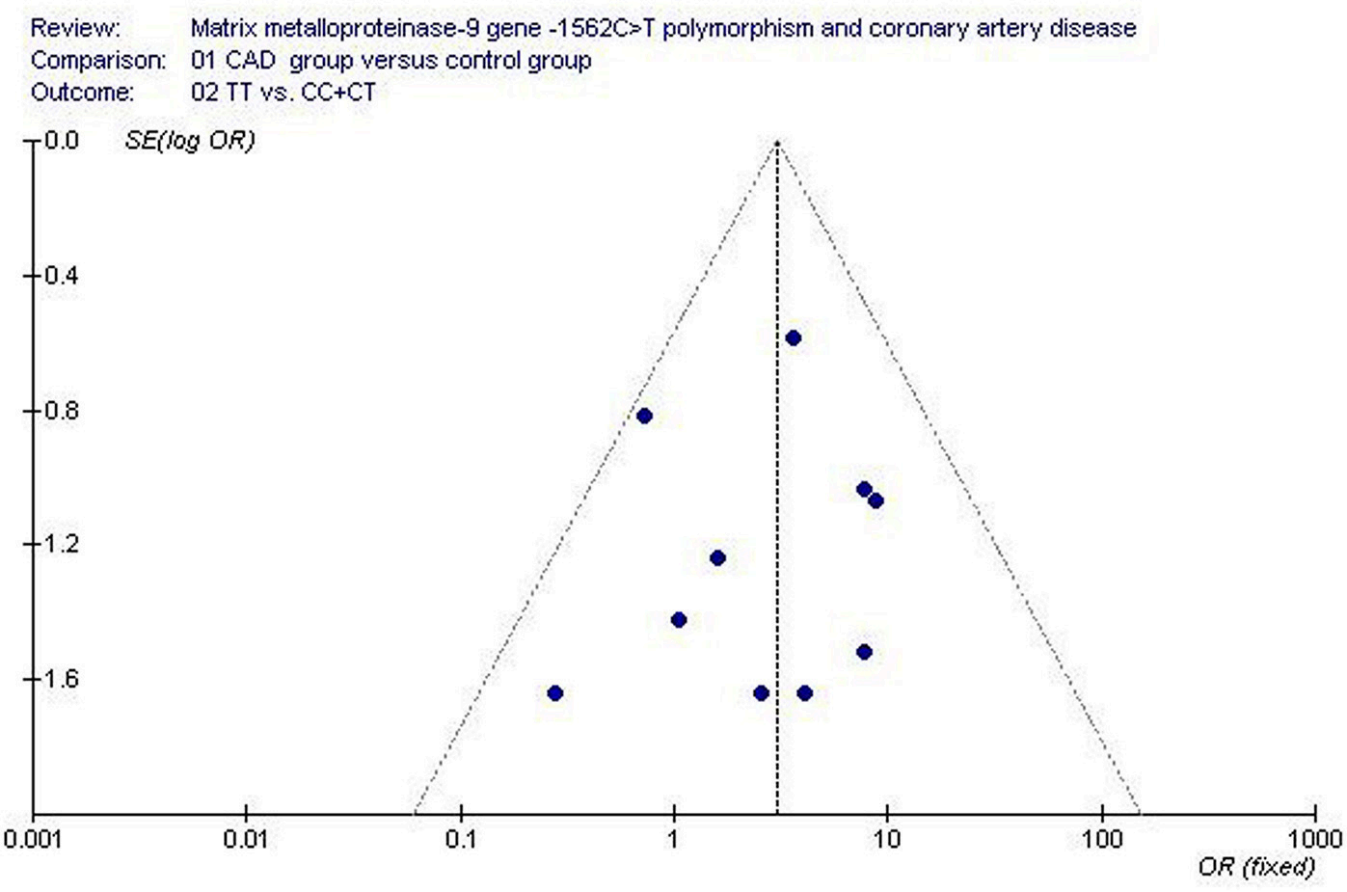
**Funnel plot for studies of the association of coronary artery disease and ***MMP-9***-1562C>T gene polymorphism under a recessive genetic model (TT vs. CC+CT)**. The horizontal and vertical axis correspond to the OR and confidence limits. OR, odds ratio; SE, standard error.

The publication bias of the studies was evaluated by funnel plot and Egger's test. There was no visual publication bias in the funnel plot (Figure 7). No statistically significant difference was detected in the Egger's test, implying no publication bias existed in the current meta-analysis under the recessive genetic model (*T* = −0.73, *P* = 0.487).

## Discussion

In the present meta-analysis, we found a significant association between *MMP-9*-1562C>T gene polymorphism and CAD in the Chinese Han population under allelic (OR: 1.60), recessive (OR: 3.05), dominant (OR: 2.23), homozygous (OR: 3.41), heterozygous (OR: 2.03), and additive genetic models (OR: 1.78). Hence, it has been concluded that in Chinese Han population, the *MMP-9*-1562C>T gene polymorphism may be associated with the increased CAD susceptibility among Han Chinese.

What contributed to the recent controversy over the association between *MMP-9*-1562C>T gene polymorphism and CAD? The meta-regression used to reveal the source of the heterogeneity detected under allelic, dominant, heterozygous and additive genetic models (P_heterogeneity_ < 0.05) suggests that patient number may have been the confounding factor. In the heterogeneity source analysis, CT1 was possibly indicated to be the main heterogeneity source under allelic and additive genetic models. Although the subgroup analysis stratified by CT1 showed a significantly increased risk of CAD in both subgroups, only one subgroup exhibited heterogeneity. Hence, CT1 was the main confounding factor contributing to the heterogeneity under the allelic and additive models. Similarly, T1 and CC0 were major confounding factors under heterozygous and dominant models, respectively. CT1, T1, CC0 may be better matched between the CAD and control groups under these genetic models.

Sensitivity analysis showed that our meta-analysis was most sensitive to the Wu et al study. Although the pooled analysis result without Wu et al study was different from the original result (Wu et al., 2009), they are still consistent in associating the presence of the gene polymorphism with an increased risk of CAD.

MMPs belong to a neutral protease family that contains zinc ions. *MMP-9* is the leading MMP expressed and secreted by the vascular cell walls. It is also secreted by monocytes, neutrophils, vascular smooth muscle cells (VSMCs), and endothelial cells. The relative molecular weight of *MMP-9* is 92KD. In its active form its molecular weight is 84KD. *MMP-9* can degrade extensive ECM substrates, including Type IV collagen, which plays a key role in the revascularization, inflammation response, and atherosclerosis progression. Research has shown an increased *MMP-9* expression level in the atherosclerotic arteries of human and animals compared to normal arteries. The *MMP-9* degradation activity was most located in the shoulder regions of plaque, the lipid core margin, and micro-vessels formation regions. This suggests that *MMP-9* may be associated with the coronary artery plaque stability and myocardial infarction (Speidl et al., 2011).

The animal experiments have discovered that the atherosclerosis lesions and VSMCs intima migration in the *MMP-9* gene knock-out mice were remarkably decreased than that in the wild-type mice (Ye, 2006). The clinical researches have confirmed that the high *MMP-9* expression level was correlated with the premature CAD, unstability of the coronary atherosclerosis plaque, the in-stent restenosis and arterial aneurysm formation (Jones et al., 2006). The prospective researches have shown that plasma *MMP-9* contents can serve as the prediction indicator for the cardiovascular diseases mortality (Blankenberg et al., 2003).

MM9 is regulated primarily at the transcriptional level. The *MMP-9*-1562C>T gene polymorphism is located in crucial regulatory elements, including the 9 bp sequence GCGCAC/TGCC (−1567 → −1559), a potential binding site for transcription inhibition proteins (Zhang et al., 1999). In 2002, Cho et al reported a change in bond zone structure and a weakened binding capacity between DNA and transcription inhibition protein, when the *MMP-9*-1562C allele was replaced by -1562T allele (Cho et al., 2002). This generates a high and low activity promoter genotype (CT/TT and CC, respectively) with increased transcription with the high activity promoter genotypes.

Increased *MMP-9* expression may contribute CAD development through a number of pathways. It may promote the VSMCs proliferation and migration, promote the injured vascular remodeling, and/or promote the plaque rupture and lead to the thrombosis, resulting in the acute coronary syndrome known as myocardial infarction (Galis et al., 2002).

Past meta-analyses on the association of *MMP-9*-1562C>T gene polymorphism and CAD (Li et al., 2012, 2013; Niu and Qi, 2012) show weaknesses in their method. Although the distribution of genes differs between populations, these meta-analyses mixed ethnic Han Chinese with other ethnicities. The present meta-analysis, on the other hand, studies only the Han Chinese population. In addition, their initial search for manuscripts was not as comprehensive as that of the current meta-analysis, making their work less objective and credible.

However, this meta-analysis is not without limitations. The large-scale studies present in the analysis were not adequate to fully elucidate the complex relationship between the *MMP-9*-1562C>T gene polymorphism and CAD. The CAD susceptibility is also influenced by environmental factors, such as smoking, diabetes, dyslipidaemia, air pollution, inflammation, and psychological factors (Wang et al., 2012; Agüero et al., 2013; Parruti et al., 2013; Wichmann et al., 2013). It is quite possible that the *MMP-9*-1562C>T gene polymorphism interact with a risk factor that was not within the scope of this study. There are also many other *MMP-9* gene polymorphisms as P574R, R+279Q, and R668Q that influence the *MMP-9* serum level (Zhi et al., 2010).

In conclusion, the current meta-analysis indicates that the *MMP-9*-1562T allele may increase the CAD risk among the Chinese population. This result has the potential to guide the therapy strategy for a CAD patient. Taking into account the limitations mentioned above, it remains necessary for these results to be verified by future studies.

## Author contributions

YL researched data. YL and CZ wrote manuscript, researched data. YL, YZ, XY, HK, and GG reviewed/edited manuscript. YL, HG, and YQ contributed to discussion, reviewed/edited manuscript. YL, JW, and XW researched data, contributed discussion.

### Conflict of interest statement

The authors declare that the research was conducted in the absence of any commercial or financial relationships that could be construed as a potential conflict of interest.
