# Supplementary material for: Matrix metalloproteinase-9 Gene-1562C>T Gene Polymorphism and Coronary Artery Disease in the Chinese Han Population: A Meta-Analysis of 5468 Subjects
Source: Front Physiol. 2016 Jun 9;7:212. doi: 10.3389/fphys.2016.00212 (PMC4899440; doi:10.3389/fphys.2016.00212)
Supplement: Supplementary file 1 [file Presentation1.PDF]

PRISMA 2009 Flow Diagram

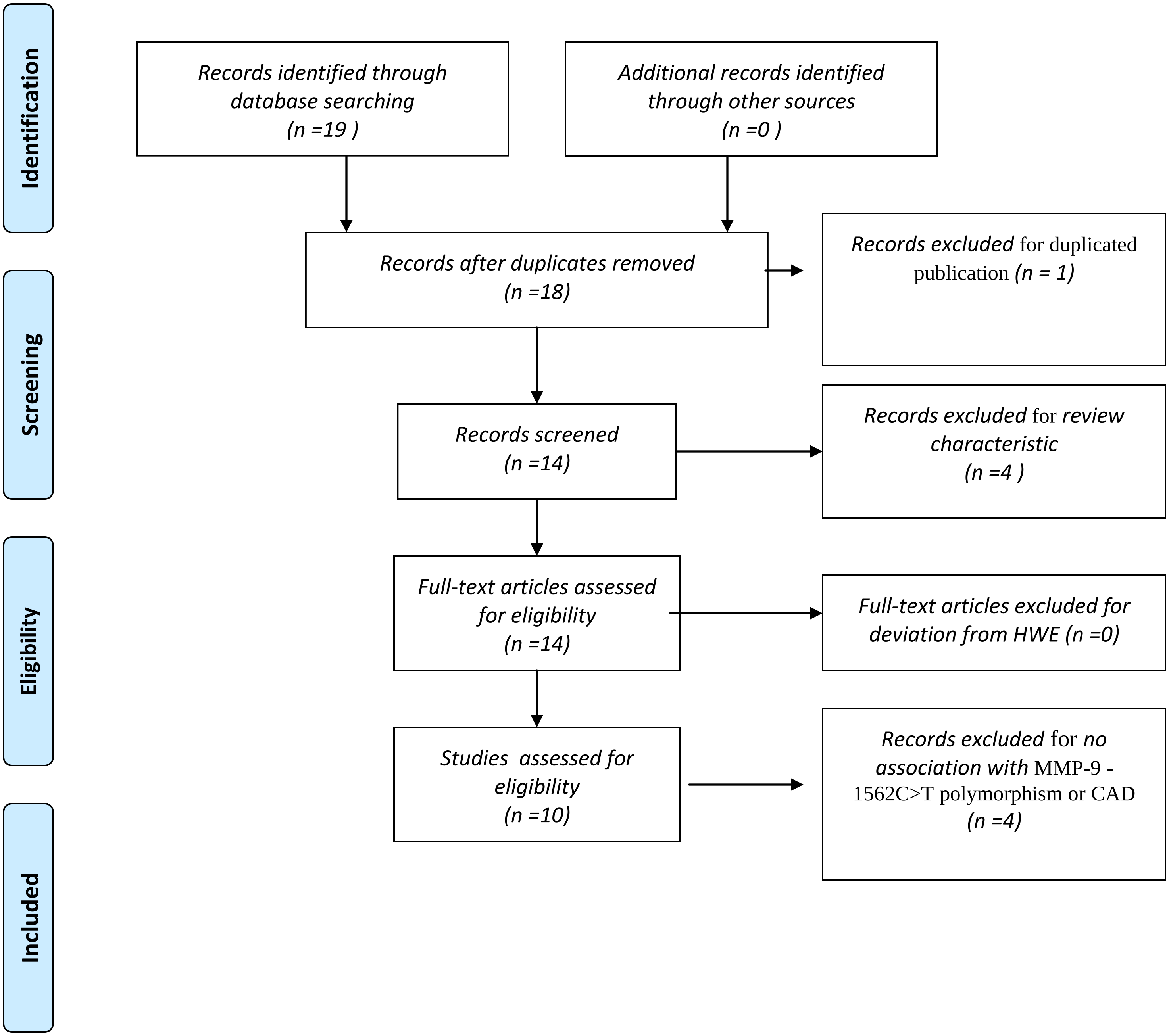

From: Moher D, Liberati A, Tetzlaff J, Altman DG, The PRISMA Group (2009). *P*referred *R*eporting *I*tems for *S*ystematic Reviews and *M*eta-*A*nalyses: The PRISMA Statement. PLoS Med 6(6): e1000097. doi:10.1371/journal.pmed1000097

For more information, visit www.prisma-statement.org.
